# Nematodes Infect, But Do Not Manipulate Digging By, Sand Crabs, *Lepidopa benedicti*

**DOI:** 10.1093/icb/icu064

**Published:** 2014-06-10

**Authors:** Meera Joseph, Zen Faulkes

**Affiliations:** Department of Biology, The University of Texas-Pan American, 1201 West University Drive, Edinburg, TX 78539, USA

## Abstract

We examined sand crabs (*Lepidopa benedicti*) for endoparasites, and found the only parasite consistently infecting the studied population were small nematodes. Because many nematodes have complex life cycles involving multiple hosts, often strongly manipulating their hosts, we hypothesized that nematodes alter the behavior of their sand crab hosts. We predicted that more heavily infected crabs would spend more time above sand than less heavily infected crabs. Our data indicate infection by nematodes was not correlated with duration of time crabs spent above sand. We also suggest that organisms living in sandy beaches may benefit from relatively low parasite loads due to the low diversity of species in the habitat.

## Introduction

Sandy beaches of oceans are a physically demanding habitat (Faulkes 2013), typically with low species diversity. Sand crabs (Decapoda, Albuneidae) are among the animals that are successful in this environment. They are specialized, obligate diggers (Faulkes and Paul 1997a, 1997b, 1998; Dugan et al. 2000) found on sandy beaches around the world (Boyko 2002). Because of concealment by sand and the scarcity of some species (Boyko 2002), the basic biology of albuneid sand crabs is poorly understood. Understanding the basic biology of albuneids includes investigating their parasites, because parasites create significant selective pressures on their hosts (Lively 1996; Agrawal 2006). As with most matters concerning sand crabs’ biology, few albuneid sand crab species have been examined for parasites, but clearly at least some do have parasites. Rhizocephalans (Boschma 1937), trematode metacercariae (Anantaraman and Subramoniam 1976), and bopyrid isopods (Markham and Boyko 1999) infect albuneid sand crabs, although none of these have been recorded on *Lepidopa*, the subject of this article.

As part of a larger set of projects designed to understand the basic biology of albuneid sand crabs (Nasir and Faulkes 2011; Murph and Faulkes 2013), we examined *Lepidopa benedicti* Schmitt 1935 for parasites. *Lepidopa benedicti* ranges from the beaches of northern Mexico to the Atlantic coastline of Southern Florida (Boyko 2002). An advantage of studying the parasites of *L. benedicti* is that it is one of the larger species in its genus, with the largest recorded individual having a carapace length of 25.3 mm (Boyko 2002). The higher upper limit in size should allow more variation in parasite load in the population (than would be true of albuneid species with a smaller maximum size) and thus more power in detecting effects of the parasite on the host. We were interested in discovering the kinds of parasites these sand crabs have, and whether those parasites might manipulate the crabs’ behavior in ways that would increase the probability of infection of subsequent hosts (parasite-induced trophic transmission [PITT]) (Lafferty 1999). Infection by acanthocephalan parasites slows the digging speed of the hippid mole crab *Emerita analoga* (Kolluru et al. 2011), which is hypothesized to make infected mole crabs more susceptible to predation by birds (Oliva et al. 2008; Kolluru et al. 2011). Thus, PITT is a plausible hypothesis for albuneid sand crabs as well. There are no documented cases of predation on *L. benedicti* in the literature, but we have observed individuals, injured during collection and thrown back into the water, to be quickly attacked by small fish in the surf. Other digging sand crabs are eaten by fish (e.g., *Albunea bulla*, Boyko 2010; *Blepharipoda occidentalis*, Lafferty 1993). This suggests that being above sand is risky for sand crabs, and parasites with another host in its life cycle would benefit from sand crabs spending more time exposed above sand.

## Materials and methods

*Lepidopa benedicti* Schmitt 1935 were collected on the beaches of South Padre Island, TX, USA, during May to August 2012, and returned to the main campus of The University of Texas-Pan American. Animals were sexed by examining the length of their pleopods (long in adult females; not noticeable in adult males), and measuring the length of their carapaces, using digital calipers. No females were ovigerous.

Individuals were video-recorded digging in a tank 300 mm wide × 150 mm deep × 200 mm high, filled with about 75 mm of sand from the South Padre Island collection site in the bottom of the tank, which was covered by seawater to a depth of about 75 mm above the sand. Video was recorded by a webcam onto a PC computer, using software (Logitech Webcam Software v. 12.10) that time-stamped the video to the nearest 0.01 s. Each individual was released at the top of the tank, and allowed to swim, unobstructed, to the bottom of the sand and submerge itself into the sand. The time spent above sand was broken into three phases: swimming (animal above sand, tailflipping, and rowing its legs; Faulkes and Paul 1997a), sitting (animal on top of sand, either stationary or moving but in such a way that it was not descending into the sand), and digging (animal actively using tail and legs to submerge in sand; Faulkes and Paul 1997a, 1997b, 1998), which were added together to calculate total time to submerge. Each individual made three digging trials, each separated by a 5-min rest period when the animal was not disturbed to minimize habituation. The average of the three trials was used for analysis.

After their behavior was recorded, individuals were dissected and parasites within them counted. Using forceps, crabs were broken into three portions, with the first break between the third pair of maxillipeds and the first pair of pereopods (chelae), and the second break between the second and third pairs of pereopods. Although we tried to divide these sections evenly, the anterior portion tended to be larger, because the carapace was more strongly attached to the anterior regions of the endophragmal skeleton.

Distribution of parasites in the body were analyzed using a one-way analysis of variance (ANOVA) and Tukey tests using Origin 7.5 (OriginLab Corporation).

Analysis of the number of parasites as a function of size, sex, and color was performed using univariate general linear model (GLM) on PASW Statistics 18 (SPSS, Inc.). A full factorial model was run withsex and color as fixed factors, and size as a covariate.

Digging times were analyzed using a multivariate GLM on PASW Statistics 18. A full factorial model was run with sex and color as fixed factors; size and number of parasites were covariates; swimming, sitting, digging, and total time above sand (sum of previous three) were dependent variables.

## Results

**Fig. 1:**
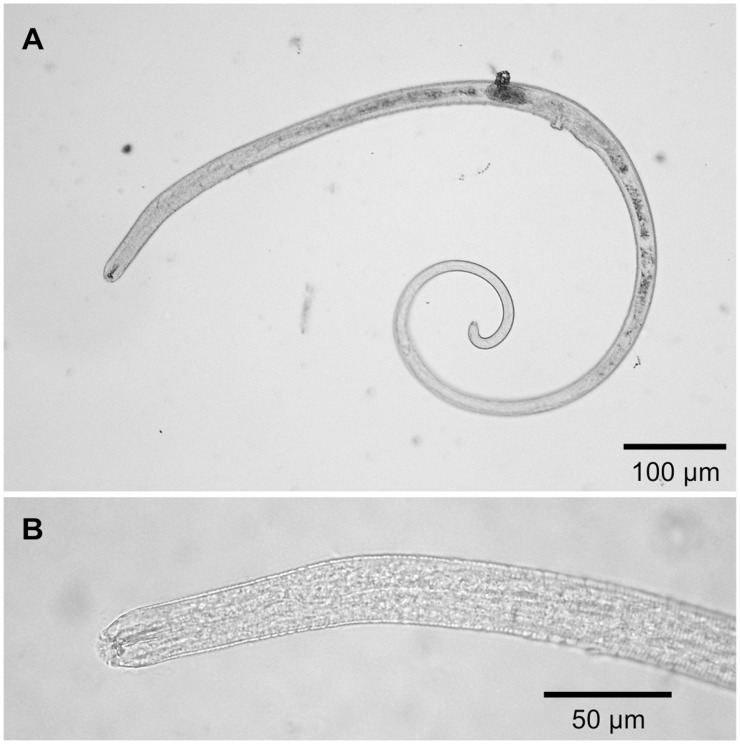
Nematode worms infecting *Lepidopa benedicti*. (**A**) Entire body and (**B**) Head. Images have been contrast-enhanced.

**Fig. 2:**
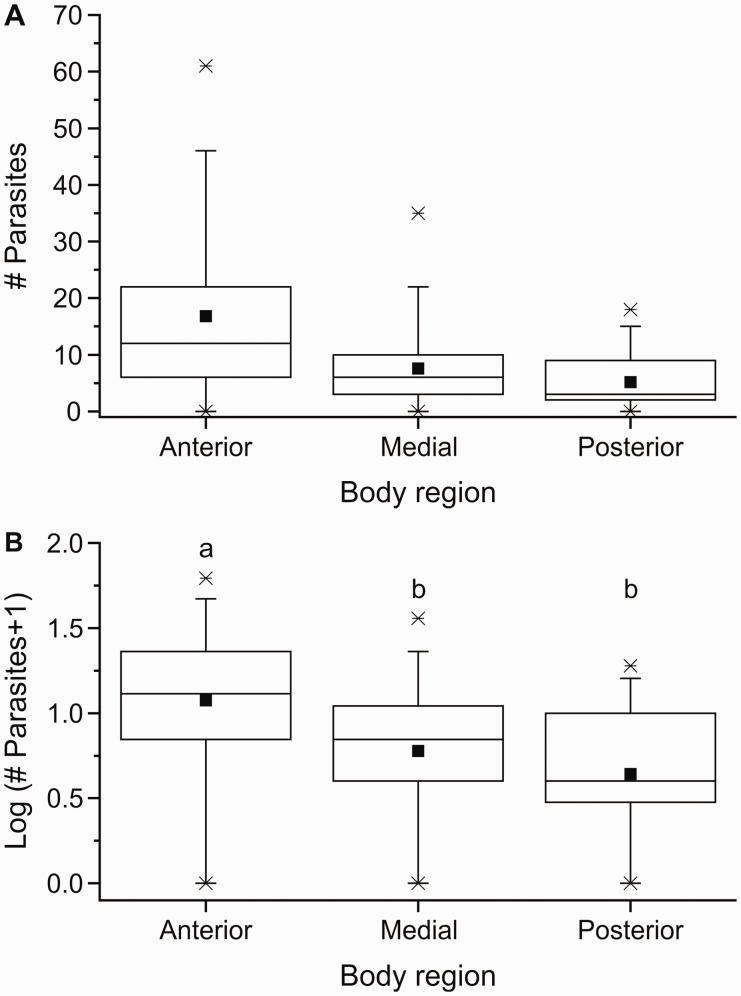
Variation of infection in different regions of the body of *Lepidopa benedicti*. (**A**) Untransformed data. (**B**) Transformed data used for statistical analysis. Boxes sharing a letter above them are not significantly different. Dot = mean; line dividing box = median; box = 50% of data; whiskers = 95% of data; asterisks = minimum and maximum.

The only parasites found in *L. benedicti* were small, immobile nematodes, approximately 1 mm long (Fig. 1). Identification of these nematodes is ongoing, but preliminary examination suggests they belong to a single species. The nematodes were definitely located in the body cavity of the sand crabs and were often found immediately under the carapace. They did not appear to be preferentially associated with any particular kind of tissue (e.g., muscle and viscera). More nematodes appeared to be in the anterior portion of crabs (Fig. 2). Because the number of parasites across body areas differed in their variance, the data were log-transformed for statistical analysis. Parasites were not evenly distributed in the three body regions (one-way ANOVA, *F* = 10.67, df = 2, 108, *P* = 0.0006): There were significantly more parasites in the anterior region, with the medial and posterior regions not differing from each other (Tukey *post-hoc* tests).

**Fig. 3:**
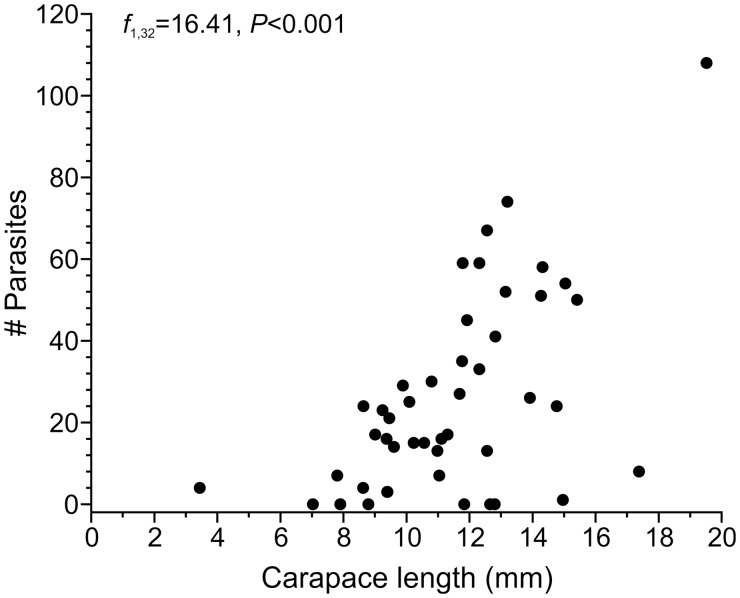
Number of nematodes is correlated with size of the host, *Lepidopa benedicti*.

**Fig. 4:**
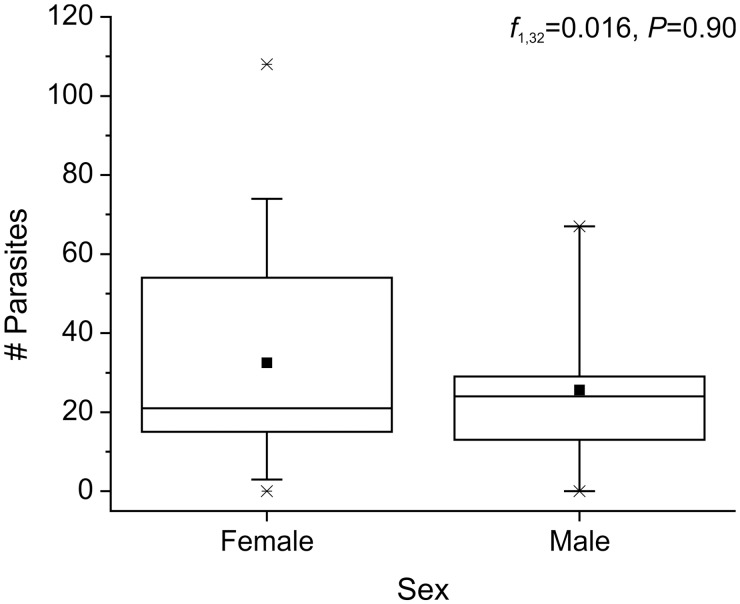
Number of nematodes is not related to the sex of the host, *Lepidopa benedicti*. Dot = mean; line dividing box = median; box = 50% of data; whiskers = 95% of data; asterisks = minimum and maximum.

**Fig. 5:**
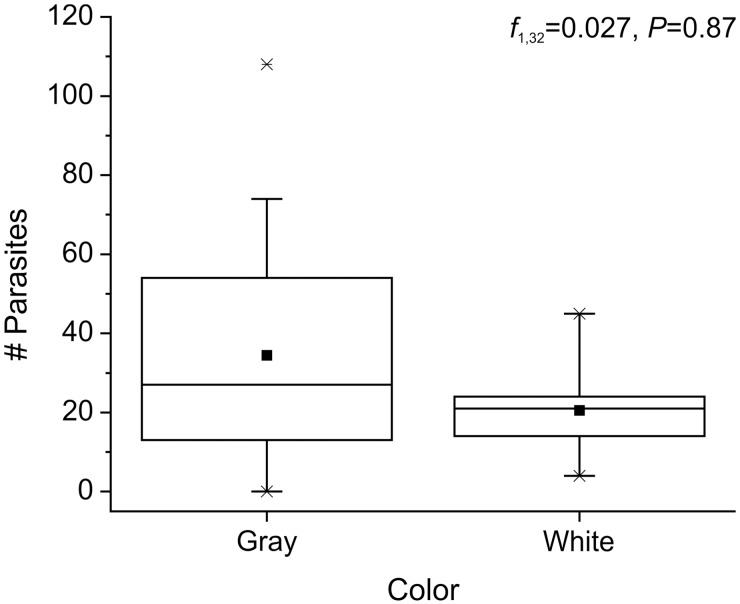
Number of nematodes is not related to color of the host, *Lepidopa benedicti*. Dot = mean; line dividing box = median; box = 50% of data; whiskers = 95% of data; asterisks = minimum and maximum.

Most *L. benedicti* (87%; 40 out of 46) were infected with nematodes, with the number of nematodes per crab ranging up to 108. The carapace length of sand crabs was significantly correlated with the number of parasites per crab (univariate GLM; *f* = 16.41, df = 1, 32, *P* < 0.001) (Fig. 3). There is a small, but significant, size dimorphism in *L. benedicti*, with females being slightly larger than males (Murph and Faulkes 2013), but there were no differences in the number of nematodes between the sexes of the *L. benedicti* hosts (univariate GLM; *f* = 0.016, df = 1, 32, *P* = 0.90) (Fig. 4). Similarly, there is a small, but significant difference in size associated with color, with gray *L. benedicti* being slightly larger than white ones (Nasir and Faulkes 2011), but there was no significant difference in the number of nematodes related to the color of the hosts (univariate GLM; *f* = 0.027, df = 1, 32, *P* = 0.87) (Fig. 5).

**Fig. 6:**
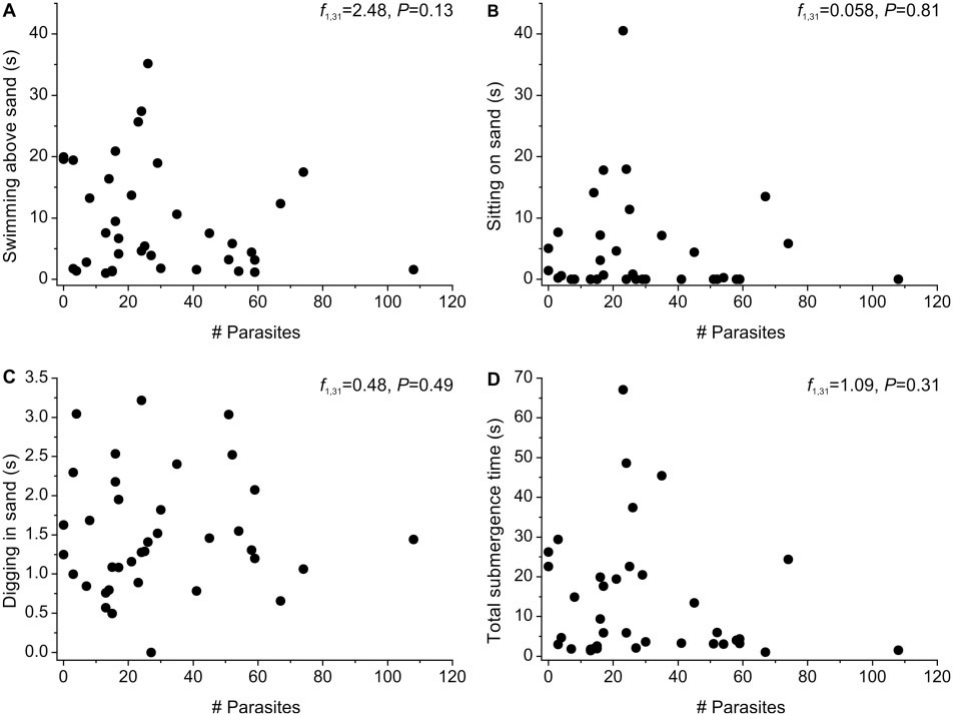
Number of nematodes is not correlated with the digging behavior of *Lepidopa benedicti* digging. (**A**) Swimming above sand by rowing legs and tailflipping. (**B**) Sitting on sand. (**C**) Digging into sand. (**D**) Total submergence time (i.e., total of A, B, and C).

**Fig. 7:**
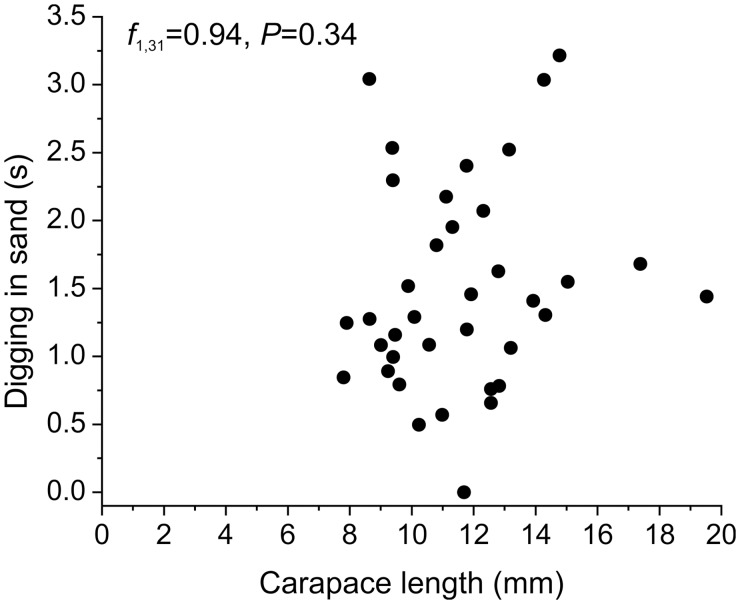
Length of carapace is not correlated with duration of digging into sand by *Lepidopa benedicti*.

There was no correlation between the number of nematodes infecting *L. benedicti* or any other variable and the duration of any aspect of the sand crabs’ digging behavior (Fig. 6 and Table 1). In *Lepidopa californica*, digging time is significantly correlated with carapace length (Dugan et al. 2000), but we found no such correlation in *L. benedicti* (*f* = 0.94, df = 1, 31, *P* = 0.34) (Fig. 7 and Table 1). We have no ready hypothesis for this difference between species; it may be due to biological differences in the species, physical differences in the type of sand at the study sites, or methodological differences in handling the animals.

**Table 1:** Size, number of parasites, sex, and color do not affect components of digging or total submergence time in *Lepidopa benedicti*

Behavior	Source	Type III sum of squares	Mean square	*f*	df	*P*-value
Swimming	Parasites	200.0335	200.0335	2.481198	1, 31	0.13
	Size	55.27048	55.27048	0.68557	1, 31	0.41
	Sex	161.1708	161.1708	1.999149	1, 31	0.17
	Color	16.73359	16.73359	0.207562	1, 31	0.65
Sitting	Parasites	3.417555	3.417555	0.057666	1, 31	0.81
	Size	19.97074	19.97074	0.336975	1, 31	0.57
	Sex	204.9692	204.9692	3.458533	1, 31	0.07
	Color	36.26839	36.26839	0.611972	1, 31	0.44
Digging	Parasites	0.294095	0.294095	0.479568	1, 31	0.49
	Size	0.575735	0.575735	0.938828	1, 31	0.34
	Sex	0.404536	0.404536	0.65966	1, 31	0.42
	Color	0.010808	0.010808	0.017625	1, 31	0.90
Total time to submerge	Parasites	259.6966	259.6966	1.087534	1, 31	0.31
	Size	66.06748	66.06748	0.276671	1, 31	0.60
	Sex	441.7061	441.7061	1.849736	1, 31	0.18
	Color	101.4747	101.4747	0.424946	1, 31	0.52

## Discussion

The only endoparasite found to infect *L. benedicti* was a small (∼1 mm) species of nematode. Its identity remains to be determined. On the one hand, it is not surprising that nematodes were the only parasites found because nematodes are famously abundant (Cobb 1915). Nematode parasites occur in sand crabs, *B. occidentalis* (Lafferty 1993, 1999), mole crabs, *E. analoga* (Lafferty 1999; Smith 2007), and other decapod crustaceans (e.g., Moravec et al. 2003). On the other hand, the high prevalence of infection in *L. benedicti* may be slightly surprising, because the diversity, prevalence, and number of parasitic nematodes in brachyuran crabs (Brattey et al. 1985; Shields 1992) and anomuran hermit crabs (McDermott et al. 2010) usually is reported as low, which may be a common trend for marine invertebrates generally (Christie 1941).

That there was no evidence of behavioral manipulation is slightly surprising. Although the life cycle of the nematode found in this study is unknown, nematode parasites of *E. analoga* have complex life cycles, in which the next host is a fish (Smith 2007), making it plausible that this species in *L. benedicti* also has a complex life cycle. Further, nematode parasites tend to be among the most effective host manipulators (Poulin 1994). The lack of manipulation may be due to the natural history of *L. benedicti*. One of the hypothesized advantages of living in sand is avoidance of predation (Faulkes 2013), and *L. benedicti* are found at low densities in this location (mode of zero individuals per 10 m transect) (Murph and Faulkes 2013), in contrast to *Emerita* species, which can have densities of several thousands of individuals per square meter of beach (Efford 1965; Perry 1980; Veloso and Cardoso 1999). Thus, the probability of trophic transmission of a parasite of *L. benedicti* may be extremely low. It is also possible that this species of nematode has a simple life cycle that does not require multiple hosts, or that nematodes affect some other behavior of their sand crab hosts. For example, nematodes may increase the probability of a sand crab emerging from the sand instead of slowing their submergence into it. Both would result in increased times spent above sand.

We hypothesize that organisms living at low density in the intertidal zone, and particularly in sandy beaches, may generally have a low parasite load. Because sandy beaches can be low in productivity, and exist at the interface between the terrestrial and marine environments, species living there may not be well suited to be hosts for parasites. First, the low productivity would affect the energy available for parasite and host alike. Second, as noted above, hosts that live there may not be well suited for a parasite with a complex life cycle that requires transmission either to terrestrial or oceanic hosts. Even if there is high prevalence of infection, as seen here, organisms living in sandy beaches may benefit from low diversity of parasites, because multiple species of parasites are disproportionately costly to resist (Koskella et al. 2012). These considerations may be overridden for species living on beaches with high population densities, such as several *Emerita* species (Efford 1965; Perry 1980; Veloso and Cardoso 1999), where the high density could create more possibilities for infection and transmission.

A next step would be to determine whether the rates of parasitic infections are the same across the entire range of this species. *Lepidopa benedicti* ranges from the Atlantic coast of southern Florida to Gulf of Mexico coast of northeastern Mexico (Boyko 2002), with individuals living on the Atlantic coast of Florida and the northern Gulf of Mexico apparently substantially larger than those living on South Padre Island (Boyko 2002; Murph and Faulkes 2013). Larger animals would provide niches for larger parasites, thereby creating the potential for greater diversity of parasitic species in large individuals of the host. Another step would be to compare the parasites within *L. benedicti* to any parasites within the sympatric mole crab, *Emerita benedicti* (Tam et al. 1996). Although these two species live in the same habitat, their mode of feeding differs: *L. benedicti* is probably a sediment feeder (Boyko 2002) and *Emerita* species are filter feeders (Efford 1966). If the nematodes infecting *L. benedicti* do so by being ingested, there may be significant differences in the parasites infecting the two species.

## Funding

This symposium was supported by the National Science Foundation Division of Integrative Organismal Systems [grant number 1338574]; the American Microscopical Society; and the Society for Integrative and Comparative Biology (Division of Animal Behavior, Division of Invertebrate Biology, and Division of Neurobiology). M.J. was supported by a Howard Hughes Medical Institute (HHMI) Science Education Grant [award number 52006321].
